# Supernormal functional reserve of apical segments in elite soccer players: an ultrasound speckle tracking handgrip stress study

**DOI:** 10.1186/1476-7120-6-14

**Published:** 2008-04-16

**Authors:** Laura Stefani, Loira Toncelli, Valentina Di Tante, Maria Concetta Roberta Vono, Brunello Cappelli, Gianni Pedrizzetti, Giorgio Galanti

**Affiliations:** 1 Sports Medicine Center, Non-invasive cardiac laboratory, University of Florence, Italy; 2 Dept. Civil and Environmental Engineering, University of Trieste, Italy

## Abstract

**Background:**

Ultrasound speckle tracking from grey scale images allows the assessment of regional strain derived from 2D regardless of angle intonation, and it is highly reproducible. The study aimed to evaluate regional left ventricular functional reserve in elite soccer players.

**Methods:**

50 subjects (25 elite athletes and 25 sedentary controls), aged 26 ± 3.5, were submitted to an echo exam, at rest and after the Hand Grip (HG) test. Both standard echo parameters and strain were evaluated.

**Results:**

Ejection fraction was similar in athletes and controls both at rest (athletes 58 ± 2 vs controls 57 ± 4 p ns) and after HG (athletes 60 ± 2 vs controls 58 ± 3 p ns). Basal (septal and anterior) segments showed similar strain values in athletes and controls both at rest (athletes S% -19.9 ± 4.2; controls S% -18.8 ± 4.9 p = ns) and after HG (athletes S% -20.99 ± 2.8; controls S% -19.46 ± 4.4 p = ns). Medium-apical segments showed similar strain values at rest (athletes S% -17.31 ± 2.3; controls S% -20.00 ± 5.3 p = ns), but higher values in athletes after HG (athletes S% -24.47 ± 2.8; controls S% -20.47 ± 5.4 p < 0.05)

**Conclusion:**

In athletes with physiological myocardial hypertrophy, a brief isometric effort produces enhancement of the strain in medium-apical left ventricular segments, suggesting the presence of a higher regional function reserve which can be elicited with an inotropic challenge and suitable methods of radial function quantification such as 2D-derived strain.

## Background

Regional myocardial function is currently evaluated by with 2D echocardiography in the four-chamber view[1] at rest and after pharmacological or physical stress. The evaluation of heart dysfunction, that is often due to ischemia, usually derives from myocardial kinetics modifications[2].

In athletes, where ischemia is an uncommon event, the main information on myocardial performance is related to global contractility, normally expressed by left ventricular Ejection Fraction (EF)[3,4].

Traditionally, a 2D echocardiographic exam is used in the four-chamber view for analysing the motion of the segments in the LV near the apex [5]; however this type of assessment can often be problematic due to the poor resolution of the lateral wall [6]. Currently the availability of new non-invasive methods for deformation parameters will make a significant contribution to a more accurate analysis of whole and segmental myocardial performance [7].

The segmental contributions of myocardial systolic function can be evaluated using non-invasive quantification of myocardial strain [8]. Strain is a physically-based measure of myocardial deformation, and it may be a tool for detecting systolic function in physiological hypertrophy [7,9,10]. Of the components of strain (longitudinal, circumferential and radial), longitudinal strain is conventionally calculated from the gradient of tissue velocity evaluated with Tissue Doppler Imaging (TDI) [9]. Estimation from TDI is limited to the segments that are well-aligned with the scan line, and it is therefore best applied to basal and medial regions [10,11]. Strain can be also estimated with "speckle tracking", a novel non-Doppler-based method that yields tissue velocity values from grey scale imaging, with high reproducibility of results [12,13] and no angle dependence. While the results of the two methods are consistent when applied to the basal segments of myocardial walls [14], only the latter allows accurate measurement of strain in the left ventricle para-apical-segment [13,15,16]. For these reasons, the regional contribution of left ventricular wall segments to overall heart performance has not yet been evaluated, especially when the medium-apical segments of the left ventricle are investigated. Experience led us to hypothesise that the efficiency of an athlete's heart during sporting activity might differ in the different LV segments, and that study of this aspect might allow us to better distinguish the various contributions of the single segments to overall heart performance. The present study thus aims to quantify the Longitudinal Peak Systolic Strain (LPSS) and Longitudinal Velocity (LV) values in a group of athletes at rest and during stress.

## Methods

For reasons of homogeneity, all the subjects studied were men. Twenty-five elite soccer players (ACF Fiorentina, Premier League) aged 26 ± 3.5, regularly trained for two hours at approximately 80% of heart rate five days a week for 10 months a year for 5 years or more, and played one game a week during the regular season. All were matched for age, weight and body surface with 25 controls who perform occasional sporting activity (Table 1).

**Table 1:** General data

**Athletes**		**Controls**	**p**
Age	26 ± 3.5	25 ± 2.6	ns
Weight Kg	75 ± 4.5	67 ± 3.5	ns
Height cm	182 ± 3.2	175 ± 3.5	ns
Body Surface m^2^	1.96 ± 0.31	1.86 ± 0.27	ns

The table shows the values of the general data of the two groups studied

All subjects gave their written consent to the study protocol and all procedures were approved by our Local Ethics Committee.

Subjects in both groups were submitted to anamnesis, including information about drugs and smoking; also to a general examination and cardiological check-up, in order to exclude family history of sudden death, hypertension, diabetes, congenital disease. All the subjects enrolled were evaluated with a standard echocardiograhic exam including systolic and diastolic parameters, as well as LPSS and LV values starting from the four-chamber view.

### Study protocol

Each subject remained at rest for 10 minutes, then was submitted to a trans-thoracic echographic exam at rest and immediately after to Hand-Grip (HG) stress. The subject held the dynamometer in one hand, squeezing the handle at 30% of their previously-established maximal grip strength for 3 minutes. The choice of employing a short, acute effort such as HG was made in order to create pressure load enhancement in the left ventricle chamber and thus in the deformation of the myocardial wall.

At rest and at the end of the HG test, heart rate (HR), systolic and diastolic blood pressure (SBP;DBP) and standard systolic and diastolic echocardiographic parameters were measured. HG was considered efficient if the mean systolic and diastolic pressure values increased significantly during handgrip as compared to the resting state (p < 0.05).

### Echocardiographic measurements

Echographic exams were performed at rest and during HG with MyLab 50 (Esaote S.p.A, Florence, Italy) equipped with a 2.5 MHz transducer. In accordance with guidelines [17], the traditional systolic function parameters (Left Ventricle Diastolic diameter (LVDd), Left Ventricle Systolic diameter (LVSd), Inter Ventricular Septum (IVS) thickness, Posterior Wall (PW) thickness, left ventricular Cardiac Mass Index (CMI g/m^2^), [18] and Ejection Fraction (EF) were calculated. Diastolic function was obtained with pulse wave transmitral Doppler flow, considering E wave velocity, A wave velocity, Iso Volumic Relaxation Time (IVRT) and Deceleration Time (DT). Left ventricle images in the four-chamber view were recorded and processed with the software package Esaote-X-Strain included in the echograph.

**Figure 1:**
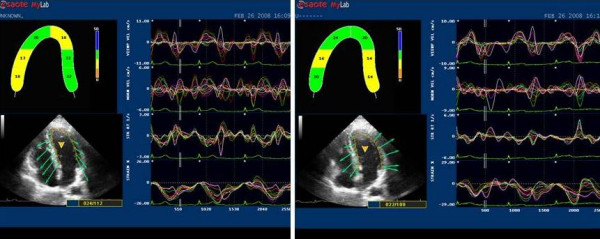
**2D LPSS evaluation in athletes' at rest (left side) and after HG (right side).** The endocardial border of the 2D left ventricular chamber is automatically followed in time frame by frame. There is no obvious differences in strain in basal and apical segments at rest, but a significant increase after HG.

**Figure 2:**
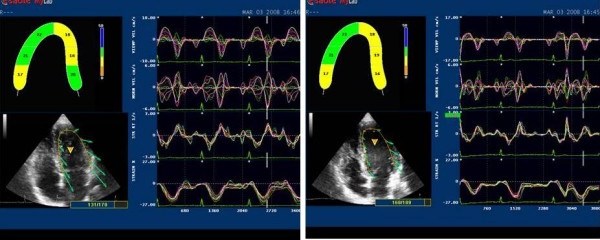
**2D LPSS evaluation at rest (left side) and after HG (right side).** Strain values show a slight but not significant increase from basal to apical segments in both situations.

This software employs the speckle tracking method to automatically evaluate myocardial dynamic properties from two-dimensional B-mode echocardiographic clips [7,8,10] (Fig 1, 2).

The software, based on a grey scale and without any angle dependence, offers the possibility of detecting strain in the basal and medium-apical segments of the left ventricle myocardial walls. Frame rate was kept between 50 and 80 Hz to optimise the application of speckle tracking for calculating Longitudinal Peak Systolic Strain (LPSS %), and LV (cm/sec) in left ventricle basal and medium-apical segments. We considered the LPSS and the LV values as the means of the values obtained at the basal segments of the interventricular septum and lateral wall and medium-apical sections of the same regions (Fig 1, 2).

### Statistical analysis

Data is presented as means ± standard deviation. The comparisons between athletes and controls at rest and at peak stress were performed with Student's two-tailed unpaired test (p < 0.05), while the comparison between basal (resting) state and Hand Grip for each group was performed with Student's paired "t" test. Statistical and power analysis were performed using Statview and STATA Stata – Corp 2003 software.

## Results

**Figure 3:**
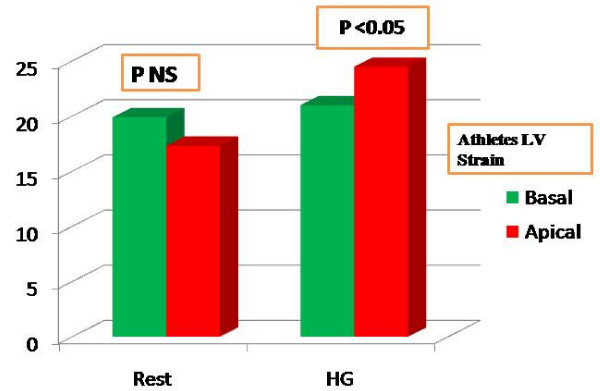
**LPSS(%) values of basal (green) and medium-apical (red) left ventricle segments in athletes at rest and after HG.** A significant increase is evident in medium-apical segments after HG. The strain values, currently negative, are conventionally expressed in positive in this graph.

**Figure 4:**
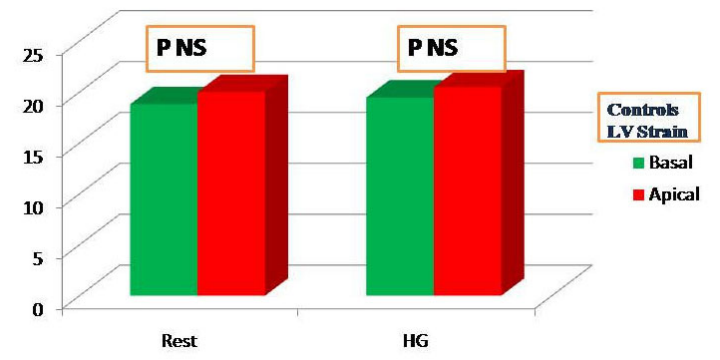
**LPSS(%) values of basal (green) and medium-apical (red) left ventricle segments in controls at rest and after HG. **No significant increase is evident at rest and after HG in the segments analyzed. The strain values, currently negative, are conventionally expressed in positive in this graph.

All the subjects were healthy, with no statistically significant differences among the general parameters (Table 1). In both groups, Systolic Pressure (SP), Diastolic Pressure (DP) and HR values increased during HG, demonstrating that the effort was efficient (Table 2). In athletes, the left ventricular dimensions were higher than in controls. However only left ventricle CMI showed a significant difference (101.54 g/m^2 athletes ^vs 92.100 g/m^2 controls ^with p < 0.05 Table 3). Left ventricle systolic and diastolic parameters including EF did not show any differences in either group at rest or after HG (Table 3). In athletes, the LPSS values of the left ventricle medium apical segments increased significantly during HG, while the control group did not show any significant increase in the same values (Table 4, 5; Fig 3, 4). Statistical power was 0.90. The LV profile in both groups maintained the physiological decrease from the basal to medium-apical segments, but only in athletes did the peak value present a statistical difference with HG (Table 4, 5). No relationship was observed between basal and apical segment LPSS values after stress and CMI in athletes (LPSS_Basal _-20.99, CMI 101.54, R = 0.09, LPSS _Medium-Apical _-24.47, CMI 101.54, R = 0,13).

**Table 2:** Haemodynamic parameters of athletes and controls at rest and after HG

**Athletes**				**Controls**		
	rest	HG	p	Rest	HG	p

HR	60 ± 2	90 ± 2	<0.05	66 ± 3	92 ± 4	<0.05
DPmmHg	80 ± 8	90 ± 3	<0.05	75 ± 4	93 ± 5	<0.05
SPmmHg	120 ± 3	140 ± 3	<0.05	125 ± 4	150 ± 5	<0.05

HR : Heart Rate; DP: Diastolic Pressure; SP : Systolic Pressure;

The hemodynamic parameters increase significantly in the two groups after HG.

**Table 3:** Echocardiographic parameters of athletes and controls

	Athletes		*P*	Controls		*p*
				
	rest	HG		rest	HG	
IVSmm	9.27 ± 0.93	9.7 ± 1	ns	8.55 ± 1.2	9.1 ± 1	ns
PWmm	9.17 ± 0.89	10.1 ± 0.8	ns	8.64 ± 1.3	9.8 ± 0.9	ns
LVEDdmm	50.37 ± 4.11	49.2 ± 3	ns	49.63 ± 4.43	48.5 ± 3	ns
LVESdmm	32.25 ± 3.53	31.4 ± 3	ns	28.45 ± 4.18	29.5 ± 3	ns
CMI gr/m2	101.54 ± 2	100.21 ± 1	ns	92.100 ± 1	91.200 ± 1	ns
LA *mm*	35.12 ± 1.2	34 ± 2.3	ns	36.10 ± 1.3	36.80 ± 1.0	ns
IVRT *ms*	81.0 ± 7.4	83.6 ± 6.4	ns	85.0 ± 4.0	86.6 ± 3.0	ns
DT *ms*	190.5 ± 13.4	178.5 ± 12.2	ns	170.4 ± 13.5	180.4 ± 12.0	ns
E/A	1.4 ± 0.3	1.33 ± 0.9	ns	1.3 ± 0.3	1.2 ± 0.7	ns
EF *%*	58 ± 2	60 ± 2	ns	57 ± 4	58 ± 3	ns

IVS interventricular septum; PW posterior wall; LVEDd Left Ventricle End Diastolic diameter; LVESd Left Ventricle End Systolic diameter; CMI Cardiac Mass Index; LA :Left Atrium; IVRT Iso Volumetric Relaxation Time; DT Deceleration Time; E/A E-wave A-wave peak velocity ratio; EF Ejection Fraction; HR Heart Rate ; SP Systolic Pressure; DP Diastolic Pressure.

The table shows the values of the main echocardiographic systolic and diastolic parameters at rest and after HG in athletes and controls. No statistical differences are evident.

**Table 4:** Longitudinal Peak Systolic Strain (LPSS) and Longitudinal Velocity (LV) in athletes

**Athletes**						
	Medium-Apical segments		*p*	Basal segments		*p*
				
	rest	HG		rest	HG	

LVcm/s	-0.93 ± 0.5	-1.40 ± 0.6	<0.05	-5.00 ± 1.6	-5.01 ± 1.3	ns
LPSS %	-17.31 ± 2.3	-24.47 ± 2.8	<0.05	-19.9 ± 4.2	-20.99 ± 2.8	ns

V(cm/s) Velocity ; LPSS% Longitudinal Peak Systolic Strain. In athletes myocardial strain and velocity values increase significantly in medium-apical segments during Hg

**Table 5:** Longitudinal Peak Systolic Strain (LPSS) and Longitudinal Velocity (LV) values in controls

**Controls**						
	Medium-Apical segments		*p*	Basal segments		*p*
				
	Rest	HG		rest	HG	

Vcm/s	-1.81 ± 0.2	-1.43 ± 1.2	ns	-5.73 ± 0.9	-4.20 ± 0.9	ns
LPSS %	-20.00 ± 5.3	-20.47 ± 5.4	ns	-18.8 ± 4.9	-19.46 ± 4.4	ns

V(cm/s) Velocity ; LPSS% Longitudinal Peak Systolic Strain. In controls, the values do not change.

## Discussion

Interest has recently grown [11,13] in evaluating the deformation parameters of heart walls in addition to the EF that normally estimates overall myocardial performance The development of the new speckle tracking software for the calculation of strain, whose application is possible in all myocardial wall sections due to its angle independence [13,15], has enhanced this interest. Commonly, the standard 2D echo exam, has several limitations, like the pitfalls in the evaluation of myocardial performance when it is focussed on the segments close to the heart apex [5,6]. On the other hand, 2D-strain evaluation offers high reproducibility of the results with mainly high-quality images.

In athletes, where a major curvature of the walls is present [3,4] as a consequence of physiological hypertrophy, the importance of estimating strain in the left ventricle medium-apical wall segment is linked to the possibility of verifying the involvement of these particular sections in overall myocardial function.

This investigation, aimed to evaluate, using LPSS, the contribution of the regional contractility of left ventricle medium-apical segments to overall heart performance in the athlete with physiological myocardial hypertrophy.

The results demonstrate the applicability of the new speckle tracking method to the calculation of strain in all sections of the heart, also in the medium -apical segments, with results which come within the normal and validated range [19] A particular effective increase in the LPSS of the left ventricle medium-apical as compared to the basal segments after an HG test is evident in athletes. HG normally produces a pressure overload in the left chamber, and the application of this study protocol enhanced the differences in LPSS values in the athletes' group, showing a gradient between the basal and medium- apical segments which was not recognizable in controls. No significant modification of EF is evident in these parameters in either groups studied after HG.

## Conclusion

These results point to the importance of considering myocardial deformation parameters in LV segments adjacent to the apex, particularly in athletes where regular training produces increased myocardial contractility. We must remember that the actual performance of the apical segments is not correctly assessable with standard 2D; also the current application of the method of strain estimation by TDI is normally limited to basal and medial wall regions due to its angle dependence.

In this study, the measurements of myocardial fibre velocity values in the same segments where strain was determined were made with the speckle tracking method, thus overcoming the limitations of aligning the beam with the scan line.

Our results show a physiological gradient with a decrease in values from basal to apical segments in the two groups, confirming the literature data

In conclusion, the significant increase in LV longitudinal strain in athletes suggests the importance of evaluating deformation parameters in order to more thoroughly assess those morphological and functional modifications in the heart which cannot be discovered by traditional echo measurements.

In agreement with the normal values recently reported in the literature [19], these results, also suggest the possibility of a routine application of these parameters in order to evaluate the athlete's heart and also to define myocardial performance in several other pathologies (involving athletes and patients alike) where heart contraction is of differing degrees according to the different wall segments.

In athletes, this evaluation might be extended in future to the investigation of the strain of the two ventricles contracting in real time, in order to determine a the different behaviour of value of these two chambers that normally work at different load pressures.

### Limitation of the study

This investigation was performed in athletes with high-quality myocardial echo-images and no further information is available about the possibility of obtaining the same results with high reproducibility in other conditions where image resolution is poor. The endocardial border needs to be optimal, with a clear delineation of the myocardial tissue and extracardiac structures.

We must also remember that myocardial contractility is a complex mechanism, recently addressed with new sophisticated software evaluating all components of wall deformation, like radial strain and torsion. In this study the evaluation of the deformation of segments around the myocardial apex, which is normally difficult from the standard 2D echocardiographic four-chamber view[1] can however be performed with a new non Doppler-derived method, speckle tracking. In addition, considering the difficulty of approaching this region using standard TDI, the investigation also included the non-Doppler velocity profile, which showed a base medium apex velocity gradient in each wall.

Although the contribution of the contractility of left ventricle medium apical segments was found to be higher than in the basal ones in soccer players submitted to an HG test, no more information about the impact of a different kind of effort, different sport or different training level is yet available. For this reason, this study must be considered as a partial investigation, which however signals the usefulness of further studies to complete performance evaluation in the athlete's heart

## Competing interests

The author(s) declare that they have no competing interests.

## Authors' contributions

The initial concept of the study was of GG and LS, who designed the study. LS, GP, LT performed all the measurements and statistical analyses. LS wrote the manuscript and all the authors contributed to, read, and approved the final version.
